# TP53 and KMT2D mutations associated with worse prognosis in peripheral T‐cell lymphomas

**DOI:** 10.1002/cam4.70027

**Published:** 2024-07-23

**Authors:** Lingling Wang, Lei Yang, Fangshu Guan, Jing Chen, Yuexin Cheng, Yuqing Miao, Jingsong He, Zhen Cai, He Huang, Yi Zhao

**Affiliations:** ^1^ Bone Marrow Transplantation Center The First Affiliated Hospital, School of Medicine, Zhejiang University Hangzhou China; ^2^ Department of Hematology The First People's Hospital of Yancheng, The Yancheng Clinical College of Xuzhou Medical University Yancheng China; ^3^ Department of Hematology The Affiliated People's Hospital of Jiangsu University Zhenjiang China

**Keywords:** KMT2D, next generation sequencing, peripheral T‐cell lymphoma, TP53

## Abstract

There are limited studies on mutation profiling for Peripheral T‐cell lymphomas (PTCL) in the Chinese population. We retrospectively analyzed the clinical and genetic landscape of 66 newly diagnosed Chinese patients. Targeted next‐generation sequencing (NGS) was performed for tissues from these patients. At least one mutation was detected in 60 (90.9%) patients, with a median number of 3 (0–7) mutations, and 32 (48.5%) cases detected with more than 4 mutations. The genes with higher mutation frequencies were TET2, RHOA, DNMT3A, IDH2, TP53, STAT3, and KMT2D respectively. When mutant genes are classified by functional group, the most prevalent mutations are related to epigenetics and signal transduction. IPI ≥2, PIT ≥2, and failure to achieve partial remission (PR) were factors for inferior progression‐free survival (PFS) and overall survival (OS). Multivariate analysis showed TP53 was an adverse factor for PFS (HR, 3.523; 95% CI, 1.262–9.835; *p* = 0.016), and KMT2D was an adverse factor for OS (HR, 10.097; 95% CI, 1.000–101.953; *p* = 0.048). Mutation profiling could help differentiate distinct types of PTCL and serve as a useful tool for determining treatment options and prognoses.

## Graphical abstract

PTCL is a rare and highly heterogeneous group of hematologic malignancies that are aggressive and characterized by a poor prognosis. Our research provides critical evidence that patients with KMT2D and TP53 mutations indicate poor outcomes. According to our observations in southern Chinese cities, mutation profiling and GEP may help differentiate distinct types of PTCL and serve as a useful tool for determining treatment options and prognoses.

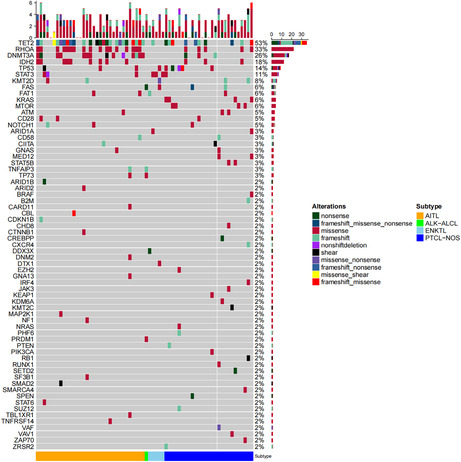

## 1 BACKGROUND

PTCL are a heterogeneous group of proliferative disorders of mature lymphocytes comprising more than 30 distinct subtypes.^1^ Although they are all derived from either post‐thymic T cells or NK cells, the disease biology and genomic features of the different subtypes vary considerably. The pathogenesis of PTCL is a complex process involving dysregulation of the TCR pathway, viral and chronic inflammation‐driven chromosomal translocations, insertions, deletions, and mutations.^2^ In addition, a wide range of epigenetic abnormalities plays a critical role in tumor chemoresistance and disease progression (PD).^3^ Except for Anaplastic Lymphoma Kinase (ALK) positive anaplastic large cell lymphoma (ALCL), patients with PTCL generally have a poor prognosis. CHOP (Cyclophosphamide, doxorubicin, vincristine, and prednisolone)‐based chemotherapy remains the “standard” treatment. In patients with CD30‐positive PTCL, Brentuximab vedotin (BV) in combination with CHP for first‐line treatment significantly improved PFS, OS, CR, and ORR compared to CHOP, with continued benefit at 5‐year follow‐up.^4^, ^5^, ^6^ In addition to CHP‐BV, most studies have focused on adding a new drug to CHOP (i.e., CHOP+X).^5^, ^7^, ^8^, ^9^ With the application of next‐generation sequencing (NGS) technology in recent years, genomics research has been widely carried out in PTCL, mainly including whole genome sequencing (WGS), exome sequencing, transcriptome sequencing analysis, genomic polymorphism analysis based on gene chip technology, and epigenetic studies.^10^, ^11^ However, there has been very little study on PTCL that covers the primary signaling pathways involved, gene expression profiling (GEP) characterization, and the prognosis of Chinese PTCL patients. By performing targeted sequencing of 103 lymphoma‐associated genes in 66 newly diagnosed PTCL patients and analyzing the correlation between high‐frequency gene mutations and clinical characteristics, we explored the mutation profiling and GEP characteristics of PTCL patients in this study.

## 2 PATIENTS AND METHODS

### 2.1 Data collection

We included 66 cases of PTCL admitted to the Bone Marrow Transplantation Center of the First Affiliated Hospital of Zhejiang University School of Medicine from July 2020 to November 2023 in this study. All cases were diagnosed as PTCL according to hematolymphoid neoplasms: the 5th edition of the WHO classification (WHO‐HAEM5). Inclusion criteria included biopsy specimens with a preliminary diagnosis and histologically confirmed PTCL, including subtypes such as extranodal natural killer (NK) TCL nasal type, Peripheral T‐cell Lymphoma, Not Otherwise Specified (PTCL‐NOS), Angioimmunoblastic T‐cell Lymphoma (AITL), and ALK‐ALCL. Immunohistochemistry mainly included CD20, CD3, CD10, BCL‐6, Ki‐67, CD5, CD30, CD2, CD4, CD8, CD7, CD56, CD21, CD23, EBER‐ISH, TCRβ, TCRδ, PD1/CD279, ALK, and TP63. Cell surface markers for flow cytometry included: CD45, CD3, CD5, CD19, CD10, CD20, CD30, CD4, CD8, CD7, CD2, TCRαβ, and TCRγδ. All pathological tissue slides were diagnosed and reviewed by two experienced pathologists. All cases were treated with first‐line therapy and were evaluated with PET‐CT or CT. Patients who did not achieve PR were switched to second‐line therapy or entered clinical trials. Exclusion criteria: patients without complete clinical data or lost to follow‐up, patients who had received radiotherapy or chemotherapy before enrollment, and patients with comorbidities of other hematological neoplasms and malignant consumptive disease. Clinical baseline data were collected from all patients at the time of initial diagnosis, including gender, age, lumbar disc herniation (LDH), Epstein–Barr virus (EBV)‐DNA, Ann Arbor stage, Eastern Cooperative Oncology Group Performance Status (ECOG PS), bone marrow biopsy, B symptoms, treatments, efficiency (complete remission [CR] or PR, stable disease [SD], or PD) and outcomes. PFS was calculated using the date of PD or death, with the latter being used to compute OS. The International Prognostic Index (IPI) and the Prognostic Index for TCL (PIT) were computed using the International Prognostic Factors in Non‐Hodgkin's Lymphoma Project system and the Intergruppo Italiano Linfomi system, respectively. All patients gave informed consent, and the study was approved by the Medical Ethics Committee of the First Affiliated Hospital of Zhejiang University School of Medicine before implementation. Furthermore, it was carried out in compliance with the Helsinki Declaration.

### 2.2 NGS sequencing

Formalin‐fixed, paraffin‐embedded tissue slices (FFPE) were used to extract genomic DNA at diagnosis. The samples were analyzed by hybridization‐capture sequencing using Illumina High‐Throughput Sequencing Platform technology, with an average sequencing depth of 16,066X and a coverage rate of >90%. The gene Pannel included exons, fusion‐associated intronic regions, and variable shear regions of 103 genes related to lymphoma, totaling about 366,802 loci. DNA sequencing was performed in all patients but RNA sequencing was done in only 30 patients, and no clinically significant fusion or mutant genes were detected. Therefore, only the results of DNA sequencing were shown in the study.

### 2.3 Treatment regimens and efficacy assessments

Fifty‐eight cases underwent imaging assessment with PET/CT and eight with CT. Thirty patients were treated with Azacitidine based on standard chemotherapy. First‐line regimens include CHOP, Dose‐adjusted EPOCH (Etoposide, vincristine, doxorubicin, prednisone, and cyclophosphamide) +/− RT, CNOP (Mitoxantrone), Cyclophosphamide + corticoids, COP (Cyclophosphamide, vincristine and prednisone), and CHOEP (Cyclophosphamide, etoposide and prednisone). Second‐line regimens include ESHAP (Etoposide, cisplatin, methylprednisolone, and cytarabine), DHAP (Cisplatin, cytosine arabinoside, and dexamethasone), GemOX (Gemcitabine, oxaliplatin, and dexamethasone) or combinations, ICE (Ifosfamide, carboplatin and etoposide), SMILE (Dexamethasone, methotrexate, ifosfamide, L‐asparaginase and etoposide), MINE (IFO, mitoxantrone and VP‐16), Bendamustine, and Auto or Allo HSCT (Hematopoietic stem cell transplant). Other therapeutic agents included XPO1 inhibitor (Selinexor), JAK inhibitor (Tofacitinib), PD‐1 inhibitor, BCL‐2 inhibitor (Venetoclax), and PI3K inhibitor (Duvelisib). One patient developed secondary AML (according to WHO‐HAEM5). One patient was treated with CD7 CAR‐T but progressed to therapy‐related myeloid sarcoma after 9 months and rapidly deteriorated to death.

### 2.4 Follow‐up

All patients were followed up until November 30, 2023, and follow‐up information was obtained by querying inpatient medical records, outpatient medical records, and telephone follow‐ups. PFS was defined as the period from the beginning of diagnosis of PTCL to PD or the time to the final follow‐up. OS was defined as the time from the diagnosis of PTCL until the patient's death or the last follow‐up.

### 2.5 Statistical analysis

SPSS 26.0 was used for data analysis, and R‐3.6.1 statistical software was used to draw heat maps. A comparison of baseline clinical characteristics of the samples was performed using the chi‐square test to calculate *p* values. OS and PFS were analyzed by the Kaplan–Meier curve analysis with the log‐rank test. Multivariate prognostic correlation analysis was performed using the Cox proportional risk regression model. *p* < 0.05 was considered a statistically significant difference.

## 3 RESULTS

Among 66 patients with PTCL, 40 were male and 26 were female, with a male‐to‐female ratio of 1.54:1. The median age was 62.5 (25–81) years, and 37 (56.1%) were >60 years. ECOG score was <2 in 61 (92.4%) patients. LDH was >250 U/L in 31 (47%) patients. Serum EBV‐DNA was positive in 38 (57.6%) cases. There were 21 (31.8%) cases with bone marrow involvement. Thirty‐nine (59.1%) cases with B symptoms. The median IPI and PTI were all two points. According to the IPI score, there were 41 (62.1%) cases in the low‐risk group and 25 (37.9%) cases in the medium‐high‐risk group. According to the Ann Arbor stage, 1 (1.5%) case was in stage I, 2 (3%) cases were in stage II, 23 (34.8%) cases were in stage III, and 40 (60.6%) cases were in stage IV. There were 4 histologic types, 33 (50%) were AILT, 1 (1.5%) was ALK‐ALCL, 5 (7.6%) were ENKTL and 27 (40.9%) were PTCL‐NOS. The response was assessed as CR in 17 (25.8%) cases, PR in 20 (30.3%) cases, SD in 7 (10.6%) cases, and PD in 22 (33.3%) cases. The median follow‐up was 16 (2–33) months, of which 16 (24.2%) died and 50 (75.8%) survived. The median OS was 28 months. The clinical characteristics of all patients are shown in Table 1.

**TABLE 1:** Baseline clinical characteristics of enrolled patients.

Characteristics	
Gender	
Male	40 (40/66,60.6%)
Female	26 (26/66,39.4%)
Age	
≤60 years	29 (29/66,43.9%)
>60 years	37 (37/66,56.1%)
With B symptoms	39 (39/66,59.1%)
IPI score	
0	2 (2/66,3%)
1	7 (7/66,10.6%)
2	32 (32/66,48.5%)
3	19 (19/66,28.8%)
4	5 (5/66,7.6%)
5	1 (1/66,1.5%)
PTI score	
0	9 (9/66,13.6%)
1	25 (25/66,37.9%)
2	26 (26/66,39.4%)
3	5 (5/66,7.6%)
4	1 (1/66,1.5%)
ECOG score	
< 2	61 (61/66,92.4%)
≥ 2	5 (5/66,7.6%)
LDH > 250 U/L	31 (31/66,47%)
Serum EBV‐DNA	
Positive	38 (38/66,57.6%)
Negative	28 (28/66,42.4%)
With bone marrow invasion	21 (21/66,31.8%)
Subtype	
AILT	33 (33/66,50%)
ALK‐ALCL	1 (1/66,1.5%)
ENKTL	5 (5/66,7.6%)
PTCL‐NOS	27 (27/66,40.9%)
Ann Arbor Stage	
I	1 (1/66,1.5%)
II	2 (2/66,3%)
III	23 (23/66,34.8%)
IV	40 (40/66,60.6%)
Number of mutant genes	
≤3	(34/66,51.5%)
>3	(32/66,48.5%)
Treatment lines	
1 line	41 (41/66, 47.1%)
2 lines	18 (18/66, 20.7%)
3 lines	5 (5/66,5.7%)
4 lines	2 (2/66,2.3%)
Treatment efficacy	
CR	17 (17/66,25.8%)
PR	20 (20/66,30.3%)
SD	7 (7/66,10.6%)
PD	22 (22/66,33.3%)
Status	
Survival	50 (50/66,75.8%)
Death	16 (16/66,24.2%)

Abbreviations: AILT, Angioimmunoblastic T‐cell Lymphoma; ALK‐ALCL, Anaplastic Lymphoma Kinase‐ Anaplastic Large cell Lymphoma; CR, Complete Remission; EBV, Epstein–Barr Virus; ECOG, Eastern Cooperative Oncology Group; ENKTL, Extranodal NK/T Cell Lymphoma; IPI, International Prognostic Index;LDH, Lumbar Disc Herniation; PD, Disease Progression; PIT, Prognostic Index for TCL; PR, Partial Remission; PTCL‐NOS, Peripheral T‐cell Lymphoma, Not Otherwise Specified; SD, Stable Disease.

**FIGURE 1:**
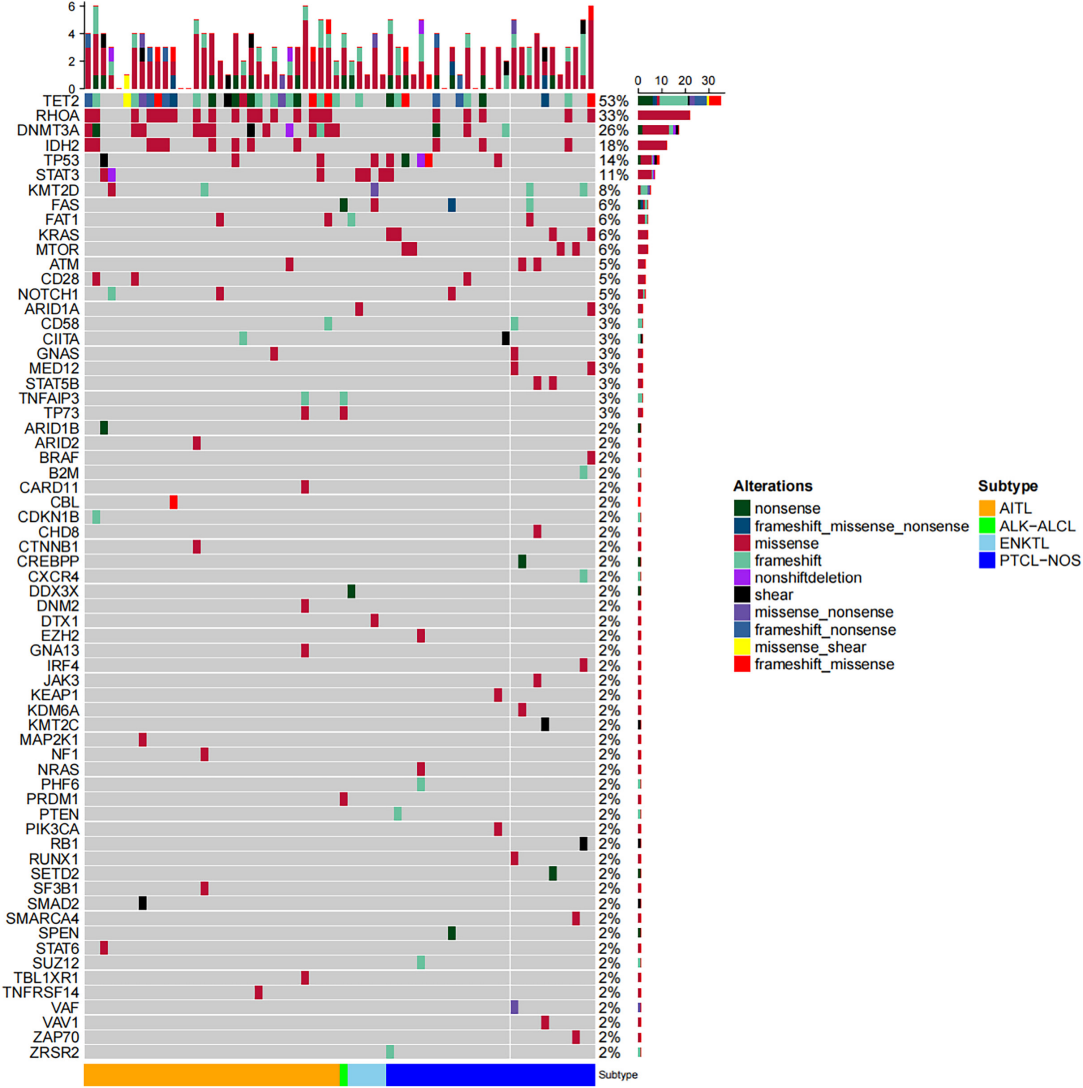
An oncoplot of all 66 patients’ genetic mutations. Every column denotes an independent patient. The number of alterations detected in each sample is shown on the top row. The type of mutation and diagnosis are represented by each row. Wild type is indicated by gray tiles. Mutations for that particular gene are represented by percent frequencies in the far‐right column.

At least 1 mutation was detected in 60 (90.9%) patients, with a median mutation number of 3 (0–7) mutations, and 32 (48.5%) cases with more than 4 mutations. The top five genes in terms of mutation rate were TET2 (53%), RHOA (33%), DNMT3A (26%), IDH2 (18%), TP53 (14%), followed by STAT3 (11%), KMT2D (8%), FAS (6%), FAT1 (6%), KRAS (6%), MTOR (6%), ATM (5%), CD28 (5%), and NOTCH1 (5%) (Figure 1). In patients with AITL, the genes with relatively high mutation frequencies were TET2 (73%), RHOA (52%), DNMT3A (42%), IDH2 (27%), STAT3 (9%), TP53 (9%), CD28 (6%), FAT1 (6%), and KMT2D (6%), NOTCH1 (6%). In the non‐AITL (mainly PTCL‐NOS) groups, the genes with comparably high mutation rates were TET2 (33%), TP53 (18%), RHOA (15%), FAS (12%), KRAS (12%), MTOR (12%), STAT3 (12%), KMT2D (9%), DNMT3A (9%), IDH2 (9%), ARID1A (6%), ATM (6%), FAT1 (6%), MED12 (6%), and STAT5B (6%). A total of 35 patients were positive for TET2, of which 22 were AITL, 9 were PTCL‐NOS, 3 were ENKTL, and 1 was ALK‐ALCL. Twenty‐two patients were positive for RHOA, of which 15 were AITL, 4 were PTCL‐NOS, 2 were ENKTL, and 1 was ALK‐ALCL. Forty‐nine patients were positive for DNMT3A, of which 31 were AITL, 12 were PTCL‐NOS, 5 were ENKTL, and 1 was ALK‐ALCL. Twelve patients were positive for IDH2, of which 10 were AITL, 2 were PTCL‐NOS, and were not detected in ENKTL and ALK‐ALCL. Nine patients were positive for TP53, of which two were AITL, six were PTCL‐NOS, 1 was ENKTL, and not detected in ALK‐ALCL. Seven patients were positive for STAT3, of which two were AITL, four were PTCL‐NOS, one was ENKTL, and not detected in ALK‐ALCL. Five patients were positive for KMT2D, of which two were AITL, three were PTCL‐NOS, and were not detected in ENKTL and ALK‐ALCL. All five KMT2D‐positive patients were positive for serum EBV‐DNA. The genes with high mutation rates and associated amino acid changes are shown in Table 2.

**TABLE 2:** Genes with high mutation rates and associated amino acid changes.

Mutant gene	Amino acid changes
TET2	p.C1273S, p.C1464Wfs*14, p.C1289Y, p.C1378Y, p.D905fs, p.E711, p.E537Rfs*30, p.G1365V, p.G1137Vfs*16, p.G1275W, p.G1361D, p.H1881P, p.H1382R, p.H1904R, p.H1781fs, p.H1893fs, p.I1873T, p.I1871Rfs*6, p.L748Ffs*6, p.L532, p.L446, p.M1907K, p.M695Nfs*17, p.N1018Mfs*15, p.N1489Mfs*82, p.N1387Ifs*62, p.N1387K, p.P929Lfs*24, p.Q622, p.Q916, p.Q731Tfs*22, p.Q1652, p.Q821, p.Q649X, p.Q958fs, p.Q1545, p.Q1654, p.Q1034, p.Q440Hfs*28, p.Q1553Lfs*19, p.Q764X, p.Q742, p.Q626Afs*11, p.Q758Rfs*55, p.Q1507X, p.Q1546X, p.R1440Tfs*38, p.R1261C, p.R544, p.R1878C, p.S1599Lfs*13, p.S714Qfs*37, p.S1290L, p.S327, p.S402, p.T665Qfs*35, p.T1122Nfs*15, p.T1093Kfs*12, p.V218Wfs*32, p.W1847, p.Y1693Sfs*20, p.Y1649Ffs*46, p.Y1631
RHOA	p.G17V
DNMT3A	p.P307L, p.Q615, p.L637R, p.R882H, p.R882S, p.R882C, p.F752I, p.S378_K382delinsR, p.P718Lfs*61, p.Y481X, p.F755S, p.D876Efs*45
IDH2	p.R172S, p.R172G, p.R172W, p.R172K, p.R172T, p.R172M
TP53	p.G105S, p.Y220C, p.Y236D, p.S215G, p.E349, p.N239_S240del, p.R209Kfs*6, p.H214P, p.C135F, p.R282P, p.E51
STAT3	p.E616K, p.D566N, p.L305_E307del, p.L312M, p.S614R, p.D427G, p.D661Y
KMT2D	p.T5464M, p.E1765Rfs*20, p.Q3831, p.E1355, p.P1465T, p.A4594fs, p.M1166Nfs*41
FAS	p.Q86, p.R250Q, p.I259T, p.D228fs, p.Q268X, p.D265G, p.D228Cfs*2
FAT1	p.I2753N, p.R3257Q, p.S3501Rfs*12, p.P1731S
KRAS	p.G13D, p.G12D, p.G12V, p.G12A
MTOR	p.S2215F, p.R2197H, p.L2209V
ATM	p.M2667T, p.Y2019C, p.E3015K
CD28	p.T195P, p.F51I
NOTCH1	p.P2514Rfs*4, p.A2548V, p.T432M

The mutations detected in these 66 patients were categorized into nine categories according to function: epigenetic, signal transduction, DNA damage response, apoptosis‐related genes, immune escape, transcription factor, cell cycle regulation, splicing factor, and transcriptional regulation, respectively. The gene mutation spectrum and frequency are shown in Table 3. The frequency of mutant genes related to epigenetics was highest at 45.78%, whereas the proportion of DNA methylation was 37.85%. These genes include DNMT3A, IDH2, and TET2. Mutant genes associated with histone methylation had a frequency of 4.67%, including EZH2, KDM6A, KMT2D, KMT2C, and SETD2. The frequency of mutant genes involved in signal transduction was 31.19%, of which 5.1% were related to the JAK–STAT pathway, including JAK3, STAT3, STAT5B, and STAT6. RAS‐MAPK pathway‐related genes accounted for 4.67%, including BRAF, CBL, KRAS, MAP2K1, NF1, and NRAS. Mutations involving the PI3K‐AKT pathway accounted for 2.8%, including MTOR, PTEN, and PIK3CA. Mutations involving the NOTCH pathway accounted for 2.3%, including DTX1, NOTCH1, and SPEN.

**TABLE 3:** The gene mutation spectrum.

Genes classified according to function		Mutant genes	Frequency (*n* = 214)
Epigenetic	DNA methylation	DNMT3A, IDH2, TET2	37.85%
	Histone methylation	EZH2, KDM6A, KMT2D, KMT2C, SETD2	4.67%
	Chromatin remodeler	ARID1A, ARID1B, ARID2, SMARCA4	2.3%
	Histone acetylation	CREBPP	0.5%
	Other Epigenetic	PHF6	0.46%
Signal transduction	JAK–STAT pathway	JAK3, STAT3, STAT5B, STAT6	5.1%
	RAS‐MAPK pathway	BRAF, CBL, KRAS, MAP2K1, NF1, NRAS	4.67%
	PI3K‐AKT pathway	MTOR, PTEN, PIK3CA	2.8%
	NOTCH pathway	DTX1, NOTCH1, SPEN	2.3%
	TCR pathway	CD28, VAV1	1.87%
	NF‐kβ pathway	CARD11, TNFAIP3	1.4%
	Cytokine receptor	CXCR4, TNFRSF14	0.9%
	WNT/β‐catenin pathway	CTNNB1	0.47%
	Other pathways	GNA13, MED12, RHOA	11.68%
DNA damage response		ATM, TP53, TP73	7%
Apoptosis related genes		FAS	2.8%
Immune escape		B2M, CD58, CIITA	2.3%
Transcription factor		DNM2, IRF4, PRDM1	1.4%
Cell cycle regulation		CDKN1B, RB1	0.9%
Splicing factor		SF3B1	0.47%
Transcriptional regulation		CHD8	0.47%
Other		DDX3X, FAT1, GNAS, KEAP1, RUNX1, SMAD2, SUZ12, TBL1XR1, VAF, ZAP70, ZRSR2	7.47%

**FIGURE 2:**
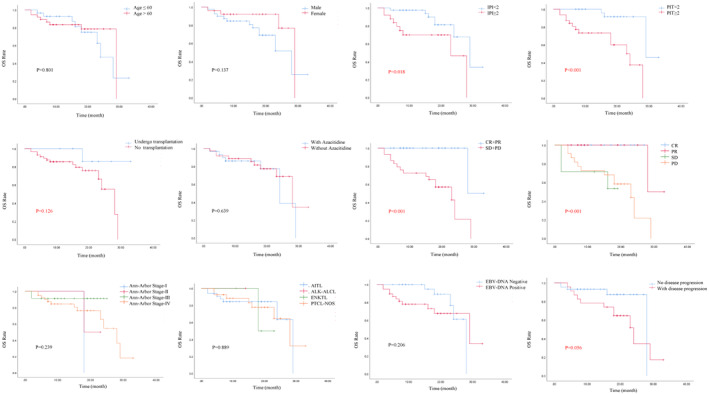
The Kaplan–Meier survival curves. IPI≥2, PIT≥2, failure to achieve PR suggested worse OS (*p* = 0.018, *p* < 0.001, and *p* = 0.001, respectively). The non‐transplanted group showed a trend toward worse OS compared to the transplanted group (*p* = 0.126).

**FIGURE 3:**
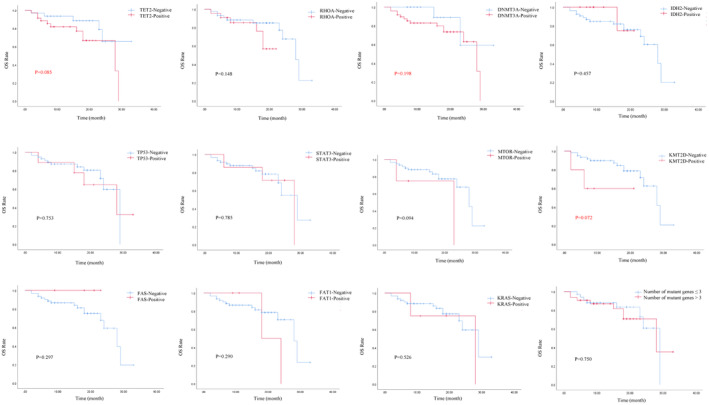
The Kaplan–Meier survival curves. TET2, DNMT3A, and KMT2D mutations suggest a tendency for poorer OS (*p* = 0.085, *p* = 0.198, and *p* = 0.072, respectively).

**FIGURE 4:**
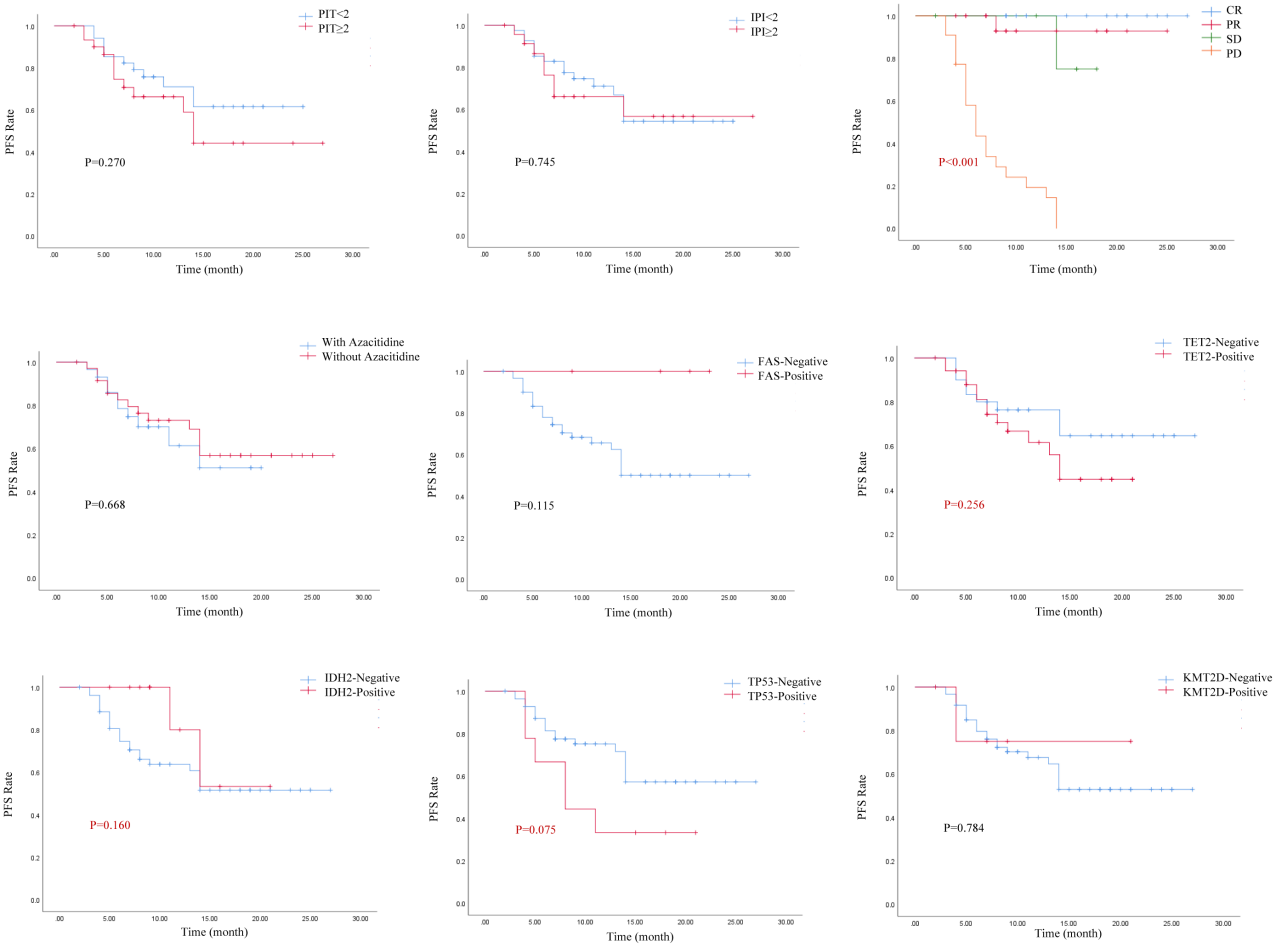
PIT≥2, failure to achieve PR, TET2 mutation, and TP53 mutation suggest worse PFS (*p* = 0.270, *p* < 0.001, *p* = 0.256, and *p* = 0.075, respectively).

IPI ≥2, PIT ≥2, and failure to achieve PR were risk factors for inferior OS (*p* = 0.018, *p* < 0.001, and *p* = 0.001, respectively). The transplant group suggested a better OS (*p* = 0.126) (Figure 2). TET2, DNMT3A, and KMT2D mutations suggest a tendency for poorer OS (*p* = 0.085, *p* = 0.198, and *p* = 0.072, respectively) (Figure 3). PIT>2, failure to achieve PR, TET2, and TP53 suggested inferior PFS (*p* = 0.270, *p* < 0.001, *p* = 0.256, and *p* = 0.075, respectively) (Figure 4). Univariate analysis suggested that gender, age, ECOG score, LDH value, serum EBV‐DNA, bone marrow invasion, B‐symptoms, Ann Arbor stage, number of mutated genes, histologic subtype, use of Azacitidine, HSCT, TET2, RHOA, DNMT3A, and IDH2 had no impact on OS and PFS (*p* > 0.05). IPI ≥2 and PIT ≥2 were factors for inferior OS (HR, 0.779; 95% CI, 0.340–1.783; *p* = 0.026 and HR, 0.594; 95% CI, 0.265–1.329; *p* = 0.004, respectively). Failure to achieve PR were factors for inferior OS and PFS (HR, 0.049; 95% CI, 0.006–0.372; *p* = 0.004 and HR, 0.002; 95% CI, 0.003–0.166; *p* < 0.001, respectively). Multivariate analyses incorporating variables with *p* < 0.1, IPI ≥2 (HR, 10.962; 95% CI, 1.118–107.528; *p* = 0.04), PIT >2 (HR, 13.664; 95% CI, 2.303–81.072; *p* = 0.004), treatment efficacy (HR, 0.007; 95% CI, 0.00–0.180; *p* = 0.003) and KMT2D mutation (HR, 10.097; 95% CI, 1.000–101.953; *p* = 0.048) were independent prognostic factors impacting OS. Treatment efficacy (HR, 0.015; 95% CI, 0.002–0.120; *p* < 0.001) and TP53 (HR, 3.523; 95% CI, 1.262–9.835; *p* = 0.016) were independent prognostic factors influencing PFS (Table 4).

**TABLE 4:** Multivariate Cox regression model for PFS and OS.

	Univariate analysis (OS)				Multivariate analysis (OS)			
	OS		PFS		OS		PFS	
Variable	HR (95% CI)	*p*‐Value	HR (95% CI)	*p*‐Value	HR (95% CI)	*p*‐Value	HR (95% CI)	*p*‐Value
Genda	2.352 (0.736–7.516)	0.149	1.444 (0.598–3.485)	0.414				
Age	1.135 (0.423–3.044)	0.802	1.598 (0.714–3.576)	0.254				
ECOG	0.371 (0.100–1.377)	0.138	0.847 (0.199–3.611)	0.823				
LDH	0.364 (0.121–1.099)	0.073	0.671 (0.300–1.499)	0.330	0.400 (0.044–3.650)	0.416		
Serum EBV‐DNA	0.504 (0.170–1.493)	0.216	0.632 (0.276–1.448)	0.278				
With bone marrow invasion	1.459 (0.397–5.359)	0.569	1.058 (0.436–2.565)	0.901				
With B symptoms	0.964 (0.354–2.624)	0.943	0.761 (0.332–1.747)	0.520				
IPI	0.309 (0.110–0.872)	**0.026**	0.779 (0.340–1.783)	0.554	10.962 (1.118–107.528)	**0.040**	2.052 (0.829–5.080)	0.120
PIT	0.108 (0.024–0.483)	**0.004**	0.594 (0.265–1.329)	0.205	13.664 (2.303–81.072)	**0.004**	1.284 (0.527–3.129)	0.582
Ann Arbor stage	0.812 (0.271–2.436)	0.711	1.348 (0.604–3.009)	0.467				
Number of mutant genes	0.851 (0.314–2.306)	0.751	0.748 (0.334–1.675)	0.480				
Subtype	1.353 (0.501–3.660)	0.551	1.237 (0.554–2.762)	0.603				
Use of Azacitidine	1.270 (0.465–3.471)	0.641	1.096 (0.481–2.495)	0.827				
Treatment efficacy	0.049 (0.006–0.372)	**0.004**	0.002 (0.003–0.166)	**<0.001**	0.007 (0.00–0.180)	**0.003**	0.015 (0.002–0.120)	**<0.001**
TET2 mutation	0.397 (0.134–1.179)	0.096	0.585 (0.256–1.339)	0.204	4.980 (0.879–28.227)	0.07	1.049 (0.414–2.658)	0.920
RHOA mutation	0.444 (0.143–1.382)	0.161	0.950 (0.404–2.233)	0.906				
DNMT3A mutation	0.388 (0.087–1.734)	0.215	0.946 (0.375–2.387)	0.906				
IDH2 mutation	2.134 (0.274–16.629)	0.469	2.653 (0.622–11.317)	0.187			0.311 (0.062–1.572)	0.158
TP53 mutation	0.825 (0.247–2.758)	0.755	0.451 (0.179–1.137)	0.091			3.523 (1.262–9.835)	**0.016**
STAT3 mutation	0.835 (0.227–3.068)	0.786	0.979 (0.291–3.296)	0.973				
KMT2D mutation	0.271 (0.059–1.255)	0.095	1.313 (0.177–9.734)	0.790	10.097 (1.000–101.953)	**0.048**		
FAS mutation	22.759 (0.003–194047.086)	0.499	23.403 (0.046–11800.115)	0.321				
FAT1 mutation	0.455 (0.101–2.045)	0.305	0.626 (0.145–2.703)	0.531				
KRAS mutation	0.611 (0.131–2.852)	0.531	0.682 (0.160–2.904)	0.604				
MTOR mutation	0.296 (0.065–1.352)	0.116	0.443 (0.101–1.939)	0.280				
HSCT	0.230 (0.030–1.788)	0.160	0.626 (0.186–2.106)	0.449				

These bolded values were *p < *0.05.

## 4 DISCUSSION

The classification of PTCL is complex, with more than 30 subtypes in the latest WHO‐HAEM5. Although pathology is the gold standard for diagnosis, it is highly susceptible to misdiagnosis due to the high degree of heterogeneity, and patients often require multiple repeat tests or consultation with experienced pathologists, resulting in a long diagnostic cycle.^12^ With the wide application of molecular biology technology, especially NGS technology, the understanding of gene mutation and pathogenesis of multiple subtypes of PTCL has been greatly improved, which provides more reference for targeted therapy.^13^, ^14^, ^15^

IPI is more commonly used in patients with NHL. In patients with PTCL, IPI showed some prognostic value for OS and PFS, but some low‐risk patients with IPI also had a poorer prognosis. PIT was similarly deficient. None of the available prognostic models provide much guidance for treatment decisions. Mutation profiles and GEP may help to distinguish different types of PTCL and identify high‐risk patients. In our study, TP53 was the only independent poor prognostic factor associated with PFS. This is similar to the fact that TP53 also suggests a poor prognosis in other hematologic malignancies.^16^, ^17^, ^18^ Genomic instability caused by TP53 alterations induces cancer chemoresistance and progression.^19^, ^20^ Several reports have shown that disruption of TP53 is associated with lymphomagenesis and treatment resistance and is increased in lymphoma relapse.^21^, ^22^, ^23^ However, the prognosis of these patients remains unfavorable even after high‐dose therapy. In our study, patients with TP53 mutations were all male. Five of them were older than 60 years old, six had B symptoms, eight were in stage III or IV, and four cases died. Three out of nine cases received HSCT and all survived. Six were combined with DNMT3A, followed by TET2 (*n* = 4), STAT3 (*n* = 3), RHOA (*n* = 2), IDH2 (*n* = 1), FAS (*n* = 1), KRAS (*n* = 1), MTOR (*n* = 1), and KMT2D (*n* = 1). Notably, although TP53 mutations are the inferior factor for PFS, but there was no statistical difference on OS. Regarding the impact of TP53 mutations on the prognosis of PTCL, our findings were consistent with the study by William T. Johnson et al. They hypothesized that novel clinical trials of noncytotoxic therapies, or performing curative allo‐HSCT allowed patients with TP53 mutations to achieve OS similar to that of TP53‐negative patients.^24^

KMT2D mutation was an independent poor prognostic factor associated with OS. Patients with KMT2D mutation were all classified as intermediate‐ to high‐risk group. 40% (2/5) patients died of PD. Four patients were treated with conventional CHOP chemotherapy in combination with oral Chidamide, three of them were evaluated as PR. The other case was treated with CHOP combined with Azacitidine, and the efficacy was assessed as SD. No patients were detected with combined TET2, RHOA, IDH2, MTOR, or KRAS mutations. Combined FAS mutations were found in 2 cases, and TP53, STAT3, and FAT1 in 1 case, separately. KMT2D co‐mutated with high‐frequency genes less often and was more likely to present as a single mutation. KMT2D is related to histone methylation. Also known as MLL2 or MLL4, it encodes histone‐lysine N‐methyltransferase, which methylates histone H3 to activate transcription. A study that included 123 EBV‐associated T or NK cell lymphoproliferative disorders (EBV + T/NK‐LPD) patients found that KMT2D mutations were detected in 12 of 87 nasal‐ENKTL. It was also the gene with the highest frequency (30.6%, 11/36) in the remaining 36 patients with ENKTL. Survival analysis suggested that KMT2D mutations in both nasal and extranasal ENKTL subgroups suggested worse OS (*p* = 0.002).^25^ KMT2D mutations likewise suggest a poor prognosis in B‐cell lymphoma.^26^ The FIL‐MCL0208 phase 3 trial showed that mutations of KMT2D and disruption of TP53 by deletion or mutation are linked to an elevated risk of progression and mortality. To our knowledge, the adverse effects of KMT2D mutations on survival in PTCL have not been documented to date. There could be two primary explanations for why KMT2D mutations did not affect survival in earlier research. First, not all KMT2D mutations fit the definition of “true” mutations because some of them are missense sequence variants that have not been reported as somatic mutations. Second, the insufficient number of NGS‐tested cases in earlier research could have resulted in an underestimation of “true” mutations.

There are very few studies on mutated genes influencing the prognosis of PTCL. Apart from the mutations in TP53 and KMT2D that we have detected, there have been reports of other genetic markers that are associated with a poor prognosis. These markers include FAT1 mutations, CD28 mutations in AITL, CDKN2A deletion in PTCL‐NOS, GATA‐3 expression, and TP63 structural abnormalities in ALK–ALCL (Table 5).^24^, ^25^, ^26^, ^27^, ^28^, ^29^, ^30^ In our study, the top five genes in PTCL were TET2 (53%), RHOA (33%), DNMT3A (26%), IDH2 (18%), and TP53 (14%). There were 63 kinds of amino acid alterations corresponding to the TET2 mutations, further validating the heterogeneity of PTCL. The genetic profile of these mutations suggests that PTCL is a complex spectrum of mutations that integrally cause events in a multistep, multi‐target oncogenic process. Analyzing the altered molecular mechanisms can provide molecular pathways and gene targets for studying the mechanisms of PTCL. We will expand the number of specimens and verify the role of these genes in PTCL at the protein level and other aspects to provide a theoretical basis for diagnosis and assessment of prognosis. In addition, given the adverse effects of TP53 and KMT2D on patient prognosis, the subsequent study at our center is to expand the size of cases and establish an independent prospective cohort for validation. We plan to incorporate the two risk factors, TP53 and KMT2D, to develop a new prognostic model to identify patients with truly high‐risk PTCL, and to explore new treatment strategies for these patients. To increase the number of patients with PTCL by enhancing WES, targeted sequencing, and RNA‐seq assays, we plan to set up multicenter research. A complete mutant genomic map of this population will be presented by the application of multi‐omics research. Create molecular stratification according to treatment response, physiological alterations, and genetic characteristics. In addition, to explore whether microenvironmental stratification can be established based on gene expression of infiltrating immune cells and stromal components.

**TABLE 5:** Summary of mutational landscape and survival outcome of PTCL.

Reference	No of patients	Median age, y (range)	Male, *n* (%)	Histology, *n* (%)	Stage III/IV, *n* (%)	Chemotherapy regimen, *n* (%)	ASCT or AlloSCT, *n* (%)	The most frequently mutated gene, *n* (%)	Variables correlating with inferior PFS in multivariate analysis	Variables correlating with inferior OS in multivariate analysis
Parrilla Castellar ER et al*, 2014* ^27^	105	51 (6–94)	70 (67)	ALK‐positive 32 (30) ALK‐negative 73 (70)	41 (39)	CHOP/CHOP‐like 73 (69.5) Other 32 (30.5)	3 (2.8)	–	DUSP22 (HR, 3.48; 95% CI, 1.13–10.69; *p* = 0.0300) TP63 (HR, 2.32; 95% CI, 1.05–5.13; *p* = 0.0380)	DUSP22 (HR, 8.63; 95% CI, 2.08–35.77; *p* = 0.0030) TP63 (HR, 4.16; 95% CI, 1.32–13.09; *p* = 0.0150)
Maura F et al*, 2021* ^25^	34	59.9 (22–85)	17 (50)	PTCL‐NOS	NA	Anthracycline induction chemotherapy 31 (91)	14 (41)	–	–	CDKN2A deletion (HR = 2.53; 95% CI: 1.006–6.3; *p* = 0.048) Age (HR = 1.06; 95% CI: 1.013–1.1; *p* = 0.013)
Laginestra MA et al*, 2020* ^26^	71	62 (14–90)	41 (57.7)	PTCL‐NOS	71	NA	NA	KMT2C (MLL3) NA, FAT1 28 (39), TET2 16 (22), KMT2D 16 (22), NOTCH1 16 (22), NOTCH2 14 (19), CREBBP 11 (16), KMT2A 8 (11), SETD2 7 (10)	–	FAT1 (*p* = 0.03)
Rohr J et al*, 2016* ^28^	105	65.5 (19–97)	61 (58)	PTCL, NOS 40 (38) AITL 53 (50) ALK‐ALCL 12 (11)	NA	NA	NA	–	–	CD28 (*p* = 0.005)
Wang T et al*, 2014* ^29^	66	64 (28–90)	34 (52)	PTCL, NOS	55 (84)	CHOP 37 (56)	NA	–	GATA‐3 (HR = 2.8; 95% CI: 1.6–5.0; *p* = 0.0005) PIT high risk (≥3 poor risk features) (HR = 2.6; 95% CI: 1.5–4.5; *p* = 0.0008)	GATA‐3 (HR = 2.0; 95% CI: 1.1–3.7; *p* = 0.02) PIT high risk (≥3 poor risk features) (HR = 3.3; 95% CI: 1.8–6.0; P = 0.0001)
William T. Johnson et al*, 2023* ^24^	132	65 (25–82)	81 (61)	PTCL‐NOS 36 (27) AITL 62 (47) PTCL‐TFH 9 (7) ALK− ALCL 15 (11) ALK+ ALCL 6 (5) MEITL 4 (3)	110 (83)	CHOEP/EPOCH 59 (45) CHOP 40 (30) BV‐CH(E)P 16 (12) CHOP‐based + novel agent 17 (13)	50 (38)	TET2 69 (52), RHOA 40 (30), DNMT3A 25 (19), TP53 21 (16), IDH2 15 (11)	Advanced‐stage disease (HR, 5.1; 95% CI, 1.1–22.5; *p* = 0.03) BM involvement (HR, 3.0; 95% CI, 1.1–8.4; *p* = 0.04) ASCT in first remission (HR, 0.3; 95% CI, 0.1–0.5; *p* < 0.001) TP53 mutations (HR, 3.1; 95% CI, 1.4–6.8; *p* = 0.005) TP53/17p deletions (HR, 4.1; 95% CI, 1.1–15.0; *p* = 0.03)	ECOG ≥2 (HR, 19.2; 95% CI, 2.8–133.0; *p* = 0.003) ASCT receipt during the first remission (HR, 0.1; 95% CI, 0.04–0.4; *p* < 0.001). CDKN2A deletion (HR, 12.1; 95% CI, 2.8–52.0; *p* < 0.001)
Yingying Ye et al, 2021^30^	49	54 (26–80)	34 (69)	AITL 33 (62) PTCL‐NOS 10 (19) ALK+ALCL 4 (8) ALK‐ALCL 6 (11)	40 (82)	CHOP‐like 37 (37/46)	7 (7/46)	TET2 34 (64), RHOA 23 (43), PCLO 12 (23), DNMT3A 10 (19)	JAK/STAT pathway (HR, 2.366; 95% CI, 0.9130–6.129; *p* = 0.0334)	TET2/TP53 (HR, 3.574; 95% CI, 1.069–11.941; *p* = 0.039)

This retrospective study, which primarily included patients from cities in southern China and where all participants underwent NGS testing before treatment, is the largest cohort of PTCL in the Chinese population reported to date. Consistent with previous studies, we confirmed that PTCL was predominantly in older male patients. No trend toward the beneficial effects of adding Azacitidine was observed in PFS and OS. Although most PTCL patients are sensitive to chemotherapy, remission durations are usually short, and recurrent disease relapses lead to poor OS. In this study, TP53‐mutant patients who received HSCT showed superior outcome benefit compared to patients who did not receive HSCT. The results of our study further suggest that HSCT is still recommended for patients with high‐risk prognostic factors to achieve better survival. However, in China, the proportion of patients undergoing HSCT after first‐line treatment is very low due to the limitations of advanced age, education, occupation distribution, and economic factors.

Although this study provides Chinese data on PTCL, there are some limitations. The patients enrolled were mainly from southern cities and the follow‐up period was still short. AITL and PTCL‐NOS constituted the majority of the histologic subtypes, with fewer types such as ENKTCL and ALCL. Regrettably, we did not perform the TBX21 assay for PTCL‐NOS in this study. Therefore, it was not possible to further analyze the subtypes of GATA3, TBX21, and TFH. Furthermore, due to the retrospective character of the study, and the patients were treated heterogeneously, the possibility of unidentified bias cannot be ruled out, and more rigorous matching studies and regression analyses are needed to exclude confounding by baseline imbalances between groups.

## 5 CONCLUSION

PTCL is a rare and highly heterogeneous group of hematologic malignancies that are aggressive and characterized by a poor prognosis. There are differences in the prognosis and gene distribution between subtypes. Based on clinical, phenotypic, and genetic characteristics, future research ought to look into the molecular pathophysiology of PTCL as well as the influence of the tumor microenvironment on treatment and drug resistance. Our study integrates NGS of 66 patients with PTCL to depict a comprehensive GEP of PTCL in Chinese patients. The TP53 mutation defines a high‐risk group with minimal response to standard or intensified treatment. Our research provides critical evidence that patients with KMT2D and TP53 mutations indicate poor outcomes. Subsequent research endeavors aim to construct molecular stratification according to genetic attributes, biological modifications, and responsiveness to treatment. To identify genuinely high‐risk patients, a novel predictive model that incorporates TP53 and KMT2D will also validated by an independent prospective cohort. According to our research, genes with higher mutation frequencies are linked to epigenetic modifications. Therefore, the use of demethylating agents in conjunction with combination immunotherapy or lower dosages of conventional chemotherapy may be taken into consideration to lessen the toxicities associated with chemotherapy in elderly, frail patients. The impact of KMT2D on the prognosis of PTCL was not addressed in earlier research. We hypothesize that this could be attributed to limited cases and insufficient genetic testing, which has left us with little knowledge of this gene.

According to our observations in southern Chinese cities, mutation profiling and GEP may help differentiate distinct types of PTCL and serve as a useful tool for determining treatment options and prognoses.

## AUTHOR CONTRIBUTIONS

**Lingling Wang:** Conceptualization (lead); data curation (lead); formal analysis (lead); funding acquisition (lead); investigation (lead); methodology (lead); project administration (lead); resources (lead); software (lead); supervision (lead); validation (lead); visualization (lead); writing – original draft (lead); writing – review and editing (lead). **Lei Yang:** Data curation (lead); formal analysis (lead); software (lead). **Fangshu Guan:** Investigation (supporting); methodology (supporting); project administration (supporting); resources (supporting). **Jing Chen:** Conceptualization (supporting); data curation (supporting); formal analysis (supporting); resources (supporting); software (supporting); supervision (supporting); validation (supporting); visualization (supporting). **Yuexin Cheng:** Conceptualization (supporting); formal analysis (supporting); writing – review and editing (supporting). **Yuqing Miao:** Conceptualization (supporting); investigation (supporting); project administration (supporting); writing – review and editing (supporting). **Jingsong He:** Conceptualization (supporting); methodology (supporting); project administration (supporting); writing – review and editing (supporting). **Zhen Cai:** Conceptualization (supporting); funding acquisition (supporting); project administration (supporting); resources (supporting); supervision (supporting); writing – review and editing (supporting). **He Huang:** Conceptualization (supporting); funding acquisition (supporting); project administration (supporting); writing – review and editing (supporting). **Yi Zhao:** Conceptualization (lead); data curation (supporting); formal analysis (supporting); funding acquisition (supporting); investigation (supporting); methodology (supporting); project administration (supporting); resources (equal); software (equal); supervision (equal); validation (equal); visualization (supporting); writing – original draft (supporting); writing – review and editing (supporting).

## ETHICS STATEMENT

The ethics committee of the First Affiliated Hospital of Zhejiang University School of Medicine authorized the study. It was performed by the ethical standards as laid down in the 1964 Declaration of Helsinki and its later amendments or comparable ethical standards. Informed consent was obtained from all patients to be included in the study.

## Data Availability

The datasets used and analyzed in the current study are available upon reasonable request from the corresponding author.
